# 15-Methacryloyloxypentadecyl Dihydrogen Phosphate Improves Resin-to-Zirconia Bonding Durability

**DOI:** 10.3290/j.jad.b3831385

**Published:** 2023-01-23

**Authors:** Zhi-cen Lu, Ling-hui Jia, Zhi-feng Zheng, Hao Yu

**Affiliations:** a PhD Candidate, Dentist, Fujian Key Laboratory of Oral Diseases & Fujian Provincial Engineering Research Center of Oral Biomaterial & Stomatological Key Laboratory of Fujian College and University, School and Hospital of Stomatology, Fujian Medical University, Fuzhou, China. Conducted the study, performed the analysis, wrote the manuscript.; b Dentist, Department of Prosthodontics, School and Hospital of Stomatology, Fujian Medical University, Fuzhou, China. Idea, hypothesis, proofread the manuscript.; c Dental Technician, Central Dental Laboratory, School and Hospital of Stomatology, Fujian Medical University, Fuzhou, China. Conducted the study, proofread the manuscript.; d Associate Professor and Associate Dean, School and Hospital of Stomatology, Fujian Medical University, Fuzhou, China; Adjunct Associate Professor, Clinic of Conservative and Preventive Dentistry, Center of Dental Medicine, University of Zürich, Zürich, Switzerland. Idea, study design, proofread the manuscript.

**Keywords:** zirconia bonding, thermocycling, 10-MDP, 15-MPDP, shear bond strength.

## Abstract

**Purpose:**

To investigate the bond durability of composite cement to zirconia after treatment with a 15-methacryloyloxypentadecyl dihydrogen phosphate (15-MPDP)-containing adhesive and 2 commercially available adhesives.

**Materials and Methods:**

Ninety zirconia bars were fabricated and bonded to prepolymerized resin composite cylinders with a composite cement after surface treatment for 20 s using the following adhesives: Adper Easy One (AEO, negative control), Single Bond Universal (SBU, positive control), and 10 wt% 15-MPDP powder mixed with Adper Easy One (15-MPDP). After storage in distilled water at 37°C for 24 h, the specimens were divided into 3 subgroups according to the aging treatment applied (n = 10): no aging treatment (0/TC), 10,000 thermocycles (1/TC), and 37,500 thermocycles (3/TC). Shear bond strength (SBS) was analyzed using two-way ANOVA (p < 0.05), and the fracture surfaces were examined under a dental microscope.

**Results:**

Significant differences in the SBSs among the surface treatments and aging treatments were observed (both p < 0.001). The 15-MPDP and SBU groups showed significantly higher SBSs than the AEO group, whereas similar SBSs were found in the 15-MPDP and SBU groups. Significant reductions in the SBSs were found after 37,500 thermocycles (p < 0.001), although no significant difference between specimens aged with 10,000 thermocycles and non-aged specimens was observed.

**Conclusions:**

The 15-MPDP-containing dental adhesive exhibited bond durability comparable to that of a well-established 10-MDP-containing universal adhesive. Aging by 10,000 thermocycles may be insufficient to disrupt the bond of composite cement to zirconia.

Yttria-stabilized tetragonal zirconia polycrystals (Y-TZPs) have become an increasingly popular choice for dental restorations.^4,17,19^ Zirconia restorations offer long-term stability in moist oral environments because of the inertness of zirconia surfaces.^21^ However, this chemical inertness makes bonding to zirconia challenging. Unlike silica-based ceramics, zirconia is not etchable with hydrofluoric acid due to the lack of a glass-ceramic phase.^20,30^ Therefore, various surface treatments have been proposed in the past few decades. These methods mainly fall into 2 categories: improving micromechanical interlocking and improving chemical bonding. Treatment with tribochemical silica followed by silane coupling agent application, or the application of universal adhesives containing functional phosphate monomers, eg, 10-methacryloyloxydecyl dihydrogen phosphate (10-MDP), are the most commonly used methods to condition zirconia surfaces.^2,11,19,26,27,30^ However, tribochemical silica coating fails to yield a uniform surface on zirconia, leaving some areas untreated. Additionally, special nanosilica-coated alumina particles may collide with the surface and prevent long-lasting adhesion,^6,11^ while priming with 10-MDP prevents these drawbacks. Therefore, 10-MDP is frequently used in primers or universal adhesives that are applied to zirconia restorations before bonding. In particular, the 10-MDP-containing universal adhesives are widely used as a surface treatment for zirconia due to their simplicity and effectiveness.^12,24^

The functional monomer 10-MDP contains a phosphate group that has been suggested to chemically interact with zirconia, while the unsaturated bonds interact with residual non-polymerized monomers of bisphenol-A-glycidyl dimethacrylate (bis-GMA). Unfortunately, the bonding performance of 10-MDP is unstable after artificial aging processes, including water storage and thermocycling.^9,17,29,33^ Therefore, modifications to 10-MDP have been proposed to improve its bonding properties, including changing the number of carbon atoms and/or ester/polyether groups.^14-16,34^ Moreover, in our previous study, alternative functional monomers were evaluated to improve the long-term chemical bond to zirconia by lengthening, shortening, or otherwise modifying the carbon chain.^7^ The results indicated that 15-methacryloyloxypentadecyl dihydrogen phosphate (15-MPDP), a modified monomer that was designed based on quantum chemical calculations, yielded more stable products than 10-MDP. Similar immediate shear bond strengths (SBSs) of zirconia were achieved using an experimental 15-MPDP-containing solution and a 10-MDP-containing solution. However, it is necessary to determine whether the bond strength to zirconia treated with 15-MPDP-containing adhesive remains stable after thermocycling. Furthermore, previous studies employed different numbers of thermocycles to evaluate the aged bond strength of composite cement to zirconia.^23^ The effects of thermocycling may differ with the number of cycles.^28^ Whether the effect of thermocycling on the composite cement-zirconia bond is dependent on the number of thermocycles has yet to be determined.

Therefore, the purpose of this study was to evaluate the durability of composite cement bonding to zirconia treated with a 15-MPDP-containing adhesive. The following null hypotheses were tested: (1) no differences exist in the bond strength to zirconia treated with different adhesives, and (2) thermocycling has no influence on the bond strength to zirconia.

## Materials and Methods

### Synthesis of 15-MPDP

15-MPDP powder was synthesized according to our previous study^7^ and examined to confirm the products obtained. ^1^H and ^31^P NMR spectra of the 15-MPDP powder were recorded using a spectrometer (JNM-ECZR, JEOL; Tokyo, Japan). Chemical shifts were recorded in parts per million (ppm, δ) relative to tetramethylsilane (SiMe_4_, δ 0.00) or the appropriate solvent (CHCl_3_, δ 7.26) as internal standards.

### Shear Bond Strength Test

Ninety Y-TZP blocks (Lava Plus, 3M Oral Care; St Paul, MN, USA) were fabricated by CAD/CAM into bars and sintered. Sintering was performed in an Austromat 674 (Dekema; Freilassing, Germany), starting at room temperature and increasing to 800°C at a rate of 12°C/min, then to 1500°C at a rate of 10°C/min, with a holding time of 2 h. After that, the samples were cooled to 1200°C at a rate of 15°C/min and then to 250°C at a rate of 20°C/min. The bars with final dimensions of 10.0 mm x 10.0 mm x 1.5 mm were polished sequentially with 400-, 800-, and 1200-grit silicon carbide abrasive paper (Matador, Starcke; Melle, Germany) and sandblasted with 50-μm aluminum oxide powder (Cobra; Renfert, Germany) at a distance of 10 mm and a pressure of 0.25 MPa.^1,19^ After ultrasonically cleaning the zirconia bars, a piece of sticky tape (thickness: 0.1 mm) with a 6-mm-diameter hole was placed on the pretreated zirconia surface. The zirconia bars were divided into 3 groups based on the surface treatments: 1. 10-MDP- and 15-MPDP-free self-etch adhesive (Adper Easy One; 3M Oral Care) (AEO, negative control); 2. 10-MDP-containing universal adhesive (Single Bond Universal, 3M Oral Care) (SBU, positive control); 3. 10 wt% 15-MPDP powder mixed with Adper Easy One (15-MPDP). The concentration of 15-MPDP was chosen based on the optimal concentration of 10-MDP.^8^ The adhesives were applied to the cementation surface for 20 s and dried with oil-free air at room temperature according to the instructions.

Resin composite (Filtek Z250, 3M Oral Care) cylinders (6 mm in diameter, 3 mm in thickness) were made in a cylindrical nylon mold and cured with a light-emitting diode curing light (Elipar S10, 3M Oral Care) at 1200 mW/cm^2^ from the 2 circular sides for 20 s each. The resin cylinders were bonded onto the prepared surfaces with 10-MDP- and 15-MPDP-free composite cement (RelyX Veneer, 3M Oral Care) under a constant load of 20 N.^9,33^ After the removal of excess cement, each specimen was light cured as mentioned above for 20 s from 4 different directions (80 s in total). Then, the tape was removed. The details of the tested materials are given in Table 1.

**Table 1:** Characteristics of the materials used in this study

Material	Main composition*	Manufacturer	Batch No.
Lava Plus	94% ZrO_2_, 6% Y_2_O_3_	3M Oral Care; St Paul, MN, USA	6701726, 7331295, 7657695, 6693759
Adper Easy One	HEMA, bis-GMA, methacrylated phosphoric esters, 1,6-hexanediol methacrylate, Vitrebond copolymer, finely dispersed bonded silica with 7-nm filler particles, ethanol, water, initiators based on camphorquinone and stabilizers	3M Oral Care	5272445
Single Bond Universal	10-MDP phosphate monomer, Vitrebond copolymer, HEMA, bis-GMA, dimethacrylate, resin, silane, ethanol, water	3M Oral Care	01127C
RelyX Veneer	TEG-DMA, bis-GMA, 66 wt% (47 vol%) zirconia/silica	3M Oral Care	NA80214
Filtek Z250	Bis-GMA, UDMA, bis-EMA, initiator (camphorquinone), 60 vol% zirconia/silica	3M Oral Care	NE46042

HEMA: 2-hydroxyethyl methacrylate; bis-GMA: bisphenol A glycidyl dimethacrylate; TEG-DMA: triethyleneglycol dimethacrylate; UDMA: urethane dimethacrylate; bis-EMA: bisphenol-polyethylene glycol dimethacrylate. *Data provided by the manufacturers.

All specimens were stored in distilled water at 37°C for 24 h and further divided into 3 subgroups according to the aging treatment performed (n = 10): 1. no thermocycling (0/TC); 2. 10,000 thermocycles (1/TC);^22,28,32^ and 3. 37,500 thermocycles (3/TC).^13,18,31^ Aging was performed in a thermocycling device (TC-501FIII, Suzhou Weier Labware; Suzhou, China) with the two water baths set to 5°C and 55°C and a dwell time of 30 s at each temperature.

The SBS test was carried out in a universal testing machine (AGS-X, Shimadzu; Tokyo, Japan) with a crosshead speed of 1.0 mm/min. The chisel-shaped rod was placed parallel to the bonded surface at a distance of 0.5 mm. The maximum load was recorded by Trapezium X software, and the SBSs were calculated according to the following formula: SBS (MPa) = maximum load (N)/area (mm^2^). Statistical analysis of SBS was performed with two-way ANOVA and Tukey’s post-hoc test. Statistical significance was set at α=0.05.

### Failure Mode Analysis

After debonding, the failure modes were assessed using a dental microscope at 16X magnification (M320, Leica; Wetzlar, Germany) and were classified as follows:^28^ adhesive failure: debonded area between the zirconia and composite cement >80%; cohesive failure: debonded area within the zirconia or composite cement >80%; and mixed failure: a combination of adhesive and cohesive failure.

## Results

**Fig 1:**
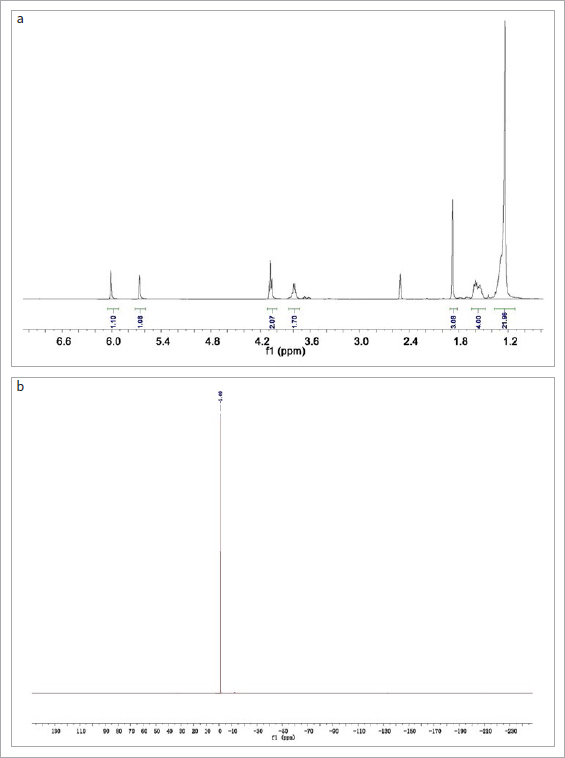
NMR spectra of 15-MPDP. a. ^1^H NMR spectrum; b. ^31^P NMR spectrum.

The ^1^H and ^31^P NMR spectra confirmed the successful preparation of the 15-MPDP powder (Fig 1).

**Fig 2:**
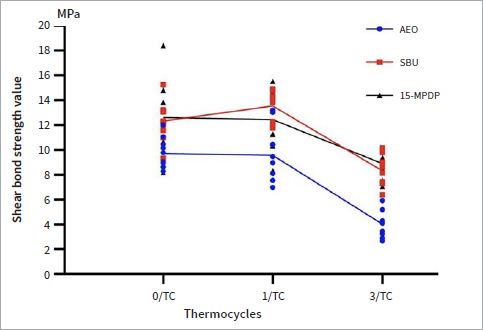
Scatter diagram showing the shear bond strengths (means and standard deviations) of all groups. AEO: Adper Easy One; 15-MPDP: 15-MPDP-based experimental adhesive; SBU: Single Bond Universal.

A scatter diagram of the SBSs from all tested groups after different surface treatment methods or aging procedures is shown in Fig 2. Means and standard deviations of the SBSs in each group are detailed in Table 2. Significant differences were found among the different surface treatments and different aging treatments (both p < 0.001). Considering the surface treatment, the groups 15-MPDP and SBU showed significantly higher SBSs than did the AEO group (both p < 0.001), regardless of the aging conditions. No significant difference was found between the groups 15-MPDP and SBU (p = 0.988). For the aging procedure, significant reductions in SBSs were found after 37,500 thermocycles (p < 0.001), although no significant difference between specimens aged with 10,000 thermocycles and nonaged specimens was observed (p = 0.777) (Fig 2).

**Table 2:** Means (SD) of the shear bond strengths determined in each of the tested groups

Surface treatment	SBS		
	0/TC	1/TC	3/TC
AEO	9.69 (1.22)^Aa^	9.56 (2.21)^Aa^	4.02 (1.27)^Ab^
15-MPDP	12.32 (1.57)^Ba^	13.54 (1.17)^Ba^	8.30 (1.21)^Bb^
SBU	12.62 (2.92)^Ba^	12.45 (2.25)^Ba^	8.90 (1.05)^Bb^

Different superscript uppercase letters within the same column indicate significant differences (p < 0.05). Different superscript lowercase letters within the same row indicate significant differences (p < 0.05). AEO: Adper Easy One; 15-MPDP: 15-MPDP-based experimental adhesive; SBU: Single Bond Universal.

**Fig 3:**
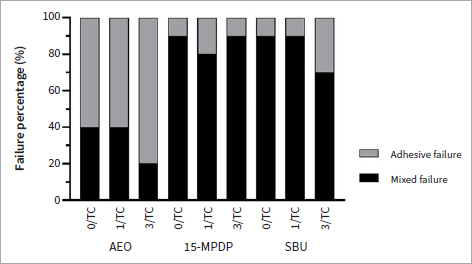
Failure modes of all tested groups. AEO: Adper Easy One; 15-MPDP: 15-MPDP-based experimental adhesive; SBU: Single Bond Universal.

**Fig 4:**
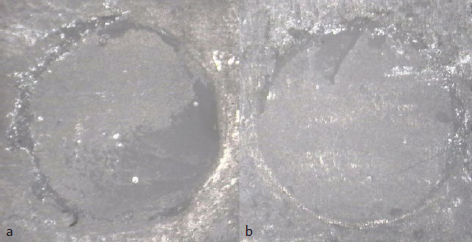
Representative failure modes of specimens from the different groups. a. Mixed failure (combination of adhesive and cohesive failure). b. Adhesive failure (debonded area between the zirconia and resin cement >80%).

Adhesive failure was observed predominantly in the AEO group, while most of the specimens in the groups SBU and 15-MPDP exhibited mixed failure (Fig 3). Representative photos of the failure modes are shown in Fig 4. A similar trend in the failure modes was observed in each surface treatment group, regardless of thermocycling.

## Discussion

Based on the current results, both null hypotheses – that there is no difference in the bond strength of zirconia subjected to different surface treatments and that aging does not affect the bonding affinity of zirconia – were rejected. The application of 15-MPDP and SBU adhesives significantly improved the bond of resin to zirconia, although the bond durability was unstable after aging. SBU was selected as a positive control, as it has been widely studied in the literature and is considered a well-established method for improving zirconia bonding. The effect of thermocycling on the bond strength of zirconia varied depending on the number of thermocycles. It is difficult to compare bond durability between different studies due to the various numbers of thermocycles selected.^11^ The majority of studies in the literature selected 4000 to 10,000 thermocycles, which is considered to be the average number that would occur in the oral cavity per year.^22^ Since 4000 to 10,000 thermocycles do not accurately represent the service life of zirconia crowns,^20^ a longer period of thermocycling was selected in this study. To simulate the thermal changes that occur in the oral cavity for as long as possible, 37,500 thermocycles (the maximum number reported in studies regarding zirconia bonding) were chosen to predict the long-term bond strength of resin to zirconia.^13^

The bond durability of 10-MDP has been well studied, but the conclusions are controversial. Some researchers have suggested that 10-MDP does not remain stable after thermocycling,^9,10^ while others hold the opposite.^20,28^ In the current study, bond strength to zirconia remained stable after 10,000 thermocycles, regardless of the surface treatment performed, which was consistent with a previous study.^25^ Interestingly, a slight increase in the bond strength was found in the group 15-MPDP after 10,000 thermocycles, indicating that potentially improved bonding performance may be achieved compared with that of group SBU. The increased bond strength may be attributed to the occurrence of post-polymerization after water storage and the higher temperature leading to additional polymerization in the resin matrix.^23^ After 37,500 thermocycles, the SBSs decreased by one-third in all groups, indicating that neither 15-MPDP-containing adhesive nor SBU would maintain a durable bond after 3 to 4 years in vivo, based on the estimation proposed by LE et al.^20^ Evidence has suggested that the MDP-Ca salts formed may hydrolyze under acidic conditions, so it can be inferred that hydrolysis may also occur on the bonding surface, corresponding to the inverse of the chemical reactions.^35^ Water diffusion into the interfacial layer may also affect bond durability.^10^ These results partially supported the idea that 10,000 thermocycles might be insufficient for testing the resin-to-zirconia bond. Nevertheless, no difference was found in the SBSs between the groups 15-MPDP and SBU after 10,000 and 37,500 thermocycles, suggesting that the experimental 15-MPDP-containing adhesive produced a bond strength of resin to zirconia similar to that of the well-established universal adhesive.

Changing the molecular structure of 10-MDP to discover other derivatives has been considered a promising approach to improve bonding to zirconia. However, few relevant studies have been performed to date. Many of the characteristics of 10-MDP, such as its flexibility, wettability, and water sorption, are influenced by spacer chains.^16^ Thus, the length of the molecule is of great importance in resin-to-zirconia bonding, and a long, hydrophobic spacer chain is recommended to promote bonding affinity.^14,16^ It has been documented that separation of the polymerizable methacrylate and phosphate groups facilitates bonding durability by preventing steric hindrance during the reactions,^7,15^ which might explain why 15-MPDP seemed to be superior to SBU after 10,000 thermocycles. Moreover, 10-MDP provides better water resistance than other monomers with more hydrophilic spacer chains.^16^ In contrast, in this study, the bond strength to zirconia in groups 15-MPDP and SBU decreased remarkably after 37,500 thermocycles. This phenomenon might be due to the mechanical stress induced by the t → m phase transformation after 37,500 cycles, although phase transformation was negligible after 10,000 thermocycles.^3,32^ In short, the addition of 15-MPDP to a dental adhesive can be considered a simplified technique to enhance resin-to-zirconia bonding.

Failure mode analysis showed that group AEO possessed a higher percentage of adhesive failures. In contrast, mixed failure predominated in the groups 15-MPDP and SBU, which was in line with our previous study.^5^

Further studies are needed to investigate the application potential of 15-MPDP for priming resin-to-zirconia bonding. In the present study, 15-MPDP was directly incorporated into a 10-MDP-free adhesive, which would simplify the implementation of this material in clinical operations. Moreover, the concentration of 15-MPDP used was determined based on our previous study.^8^ This presents the possibility of enhancing bond strength by changing the concentration of 15-MPDP or altering the solvent.

## Conclusions

Compared with the well-established 10-MDP-containing adhesive, the 15-MPDP-containing adhesive exhibited similar bonding performance after thermocycling. Aging by 10,000 thermocycles may be insufficient to demonstrate a negative effect on resin-to-zirconia bonding.

## Clinical relevance

The addition of 15-MPDP to dental adhesive is considered a simplified technique to enhance resin-to-zirconia bonding.
